# Clinical and Laboratory Predictors of Bone Mineral Density Trajectories and Osteoporosis Progression: A Retrospective Longitudinal Cohort Study

**DOI:** 10.3390/jcm15176581

**Published:** 2026-08-26

**Authors:** Layal K. Jambi, Saeed M. Kabrah

**Affiliations:** 1 Radiological Sciences Department, College of Applied Medical Sciences, King Saud University, P.O. Box 11451, Riyadh 4545, Saudi Arabia; 2 Department of Clinical Laboratory Sciences, Faculty of Applied Medical Sciences, Umm Al-Qura University, P.O. Box 50199, Makkah 21955, Saudi Arabia; smkabrah@uqu.edu.sa

**Keywords:** bone mineral density (BMD), dual-energy X-ray absorptiometry (DXA), biomarkers, vitamin D

## Abstract

**Background:** Whether routinely available laboratory measures are associated with longitudinal bone mineral density (BMD) change in clinical practice remains uncertain, particularly when treatment exposure and other major skeletal determinants are incompletely recorded. This study examined BMD trajectories and incident osteoporosis in a Saudi dual-energy X-ray absorptiometry (DXA) cohort. Anti-osteoporosis therapy, glucocorticoid exposure and menopausal status were unavailable, limiting causal interpretation. **Methods:** This retrospective longitudinal cohort included DXA examinations performed at King Saud University Medical City (KSUMC), Riyadh, from 2016 to 2021. The trajectory analysis comprised 2147 patients with at least two scans and a minimum one-year interval. Diagnostic category was based on the lowest T-score across the lumbar spine, bilateral femoral necks and distal radius. Twenty linear mixed-effects (LME) models evaluated time-by-biomarker interactions with Benjamini–Hochberg correction. Cox proportional hazards (PH) regression was the primary analysis of incident osteoporosis among 789 patients without osteoporosis at baseline. **Results:** During 2045 person-years of observation, 107 patients developed osteoporosis (5.2 events per 100 person-years). No event occurred among 124 patients with normal baseline BMD, compared with 107 events among 665 patients with baseline osteopenia (log-rank *p* < 0.001). In the complete-case Cox model (n = 384; 53 events; C-index = 0.679), baseline osteopenia had a hazard ratio (HR) of 3.12 (95% Confidence interval (CI) 0.89–10.97; *p* = 0.076); no modelled covariate reached statistical significance. None of the 20 time-by-biomarker interactions remained significant after multiplicity correction (smallest q = 0.214). Four nominal terms with raw *p* < 0.10 were below the measurement-based clinical threshold and were compatible with chance variation. **Conclusions:** No routinely measured biomarker demonstrated clinical utility for identifying BMD trajectory in this cohort. Baseline DXA category separated the observed progression pattern, although this partly reflects the distance from the diagnostic threshold and should not be interpreted as a validated prediction model. The principal contribution is a carefully characterised null result. Prospective studies with explicit treatment tracking and repeated bone-turnover measurements are required for confirmation.

## 1. Introduction

Osteoporosis causes fragility fractures with substantial morbidity, mortality and cost. Saudi Arabia faces a rising burden driven by population ageing, widespread vitamin D insufficiency and a high prevalence of metabolic disease [1,2,3].

Routine laboratory measures are often obtained alongside DXA, and each has a plausible mechanistic link to bone. Reduced renal function impairs 1α-hydroxylation of 25-hydroxyvitamin D (25-OH vitamin D) and drives secondary hyperparathyroidism through phosphate retention and fibroblast growth factor-23 excess, so Estimated glomerular filtration rate (eGFR) is a candidate marker of mineral-metabolism disturbance [4]. Anaemia reflects marrow function and chronic inflammation, both of which influence osteoblast–osteoclast balance [5]. Thyroid hormone excess accelerates remodelling, making Thyroid-stimulating hormone (TSH) a candidate marker of turnover [6]. Ferritin has been linked to bone through iron-mediated oxidative stress, though it is also an acute-phase reactant [7]. Each of these links is indirect: none of these analytes measures bone remodelling itself, all are strongly influenced by the acute clinical circumstances that prompted the test, and none has an established threshold at which skeletal risk changes. This is the central reason such markers may perform poorly as predictors in routine data even where the underlying biology is real.

Mendelian randomisation studies using 143 genetic instruments in over 417,000 participants have found no causal effect of circulating 25-hydroxyvitamin D on BMD at any skeletal site across the lifespan [8], which sets a low prior expectation for vitamin D as a trajectory predictor. In type 2 diabetes mellitus (T2DM), areal BMD is typically preserved or elevated despite raised fracture risk, because advanced glycation end-products and impaired collagen cross-linking degrade bone quality without reducing bone mass [9,10]. Trabecular bone score (TBS) would in principle address this quality–quantity gap [11], but it was not available in this dataset and is therefore mentioned only among future directions rather than as a study variable.

This study addresses two related but distinct questions. First, do baseline laboratory measures modify the rate of site-specific BMD change? The outcome is the site-specific BMD value over time and the analysis is a linear mixed-effects model with a time × biomarker interaction term. Second, do baseline clinical variables predict progression to osteoporosis among patients who do not have it at baseline? The outcome is time to the first DXA meeting the osteoporosis threshold and the primary analysis is Cox proportional hazards regression. The first question concerns a continuous process, the second a clinical transition; they are reported separately throughout.

## 2. Materials and Methods

### 2.1. Study Design and Setting

Retrospective longitudinal cohort study of all DXA examinations performed at King Saud University Medical City, Riyadh, between 18 December 2016 and 30 December 2021. Reporting follows Strengthening the Reporting of Observational Studies in Epidemiology (STROBE) [12] (Supplementary Table S1). The study was approved by the Institutional Review Board approval: King Saud University, reference E-22-6548, 9 January 2022. Patient identifiers were pseudonymised before analysis; the research team had no access to direct identifiers. The requirement for individual informed consent was waived because the study was retrospective, non-interventional, and based on de-identified routinely collected clinical data.

### 2.2. Data Source, Extraction and Audit

DXA results are stored as free-text radiologist narratives in the institutional radiology information system. Values were extracted with a pre-specified regular-expression algorithm applied to normalised text, parsing each skeletal site independently. The extraction code, the regular expressions, and the audit worksheet are provided as Supplementary Table S2. Auditing proceeded in two stages. First, a random sample of 100 reports was abstracted manually by a reader blinded to the algorithm output, and agreement was assessed against the exact numeric value rather than a tolerance band: a record counted as agreeing only when the extracted BMD matched the manually abstracted BMD to three decimal places. On this exact-match criterion agreement was 98.0% for the lumbar spine, 97.0% for the left femoral neck, 96.0% for the right femoral neck and 94.0% for the radius; every discordant record arose from non-standard narrative formatting rather than numeric error. Second, an internal consistency check compared each parsed BMD against its parsed T-score for the same site; records failing this check were re-parsed. This audit identified a pipeline defect in which the first femoral neck value had been propagated to all non-lumbar sites, and a parallel defect affecting site-specific T-scores. Both were corrected by re-parsing all 13,626 reports before any inferential model reported here was fitted.

Densitometry was performed on a Hologic Discovery A (S/N 84291) fan-beam densitometer running APEX software version 4.5.3 (Hologic Inc., Marlborough, MA, USA), with daily spine-phantom quality control throughout the study period. The institution-specific long-term precision error for lumbar spine BMD, calculated as the root-mean-square coefficient of variation from 30 patients scanned in duplicate per ISCD methodology, is 1.2% (0.012 g/cm^2^), giving a least significant change (LSC) at 95% confidence of 0.033 g/cm^2^.

### 2.3. Skeletal Sites and Diagnostic Classification

Four sites are reported by this institution: lumbar spine (L1–L4), left femoral neck (FN-L), right femoral neck (FN-R) and distal radius. Total hip BMD was not acquired. The institutional DXA protocol during the study period used a dual-femur neck-only acquisition, and total hip regions of interest were not analysed or reported; only 4 of 13,626 narratives (0.03%) referenced a total hip result. Total hip BMD therefore could not be included, and this is a genuine limitation because the total hip is the most reproducible site for monitoring and the reference site for the Fracture Risk Assessment Tool (FRAX). Following World Health Organization (WHO) criteria and ISCD guidance [13,14], osteoporosis was defined as a T-score of −2.5 or lower at the lowest of the four measured sites, osteopenia as a lowest T-score above −2.5 but at or below −1.0, and normal as a lowest T-score above −1.0. Site-specific classification counts are given in Table 1.

### 2.4. Trajectory Cohort Definition

Patients with two or more DXA examinations were eligible for trajectory analysis. A minimum inter-scan interval of one year was required. The rationale is precision: with a least significant change of 0.033 g/cm^2^, an interval of a few weeks cannot distinguish true change from measurement error, and annualising such an interval multiplies that error by a large factor, producing implausibly extreme slopes. Of 2318 patients with serial scans, 30 (1.3%) had a total span shorter than six months and 159 (6.9%) shorter than one year. Applying the one-year threshold yielded 2147 patients and reduced the number with an implausible absolute slope above 0.10 g/cm^2^/yr from 57 to 33. Results using no minimum interval and a six-month minimum are reported in Supplementary Table S3 and did not differ materially in direction or significance.

Per-patient annual change was estimated by ordinary least-squares regression of lumbar spine BMD on time. Strata were stable or improved (slope ≥ −0.005 g/cm^2^/yr), moderate loss (−0.020 to −0.005) and rapid loss (<−0.020). These cutoffs were chosen so that the moderate/rapid boundary approximates half the least significant change per year (0.0165 g/cm^2^/yr), meaning a patient classed as rapid loss would exceed the least significant change within approximately two years. Because the cutoffs remain partly conventional, three alternative schemes were tested; stratum sizes shifted as expected but no between-stratum comparison in Table 2 changed its significance status, and full results appear in Supplementary Table S4.

### 2.5. Laboratory Data, eGFR and Covariates

For each DXA visit the most recent laboratory result within the preceding 180 days was linked. Haemoglobin is reported in g/L, the source laboratory’s unit. Estimated glomerular filtration rate was calculated with the 2021 race-free CKD-EPI (Chronic Kidney Disease Epidemiology Collaboration) equation after converting creatinine from µmol/L to mg/dL by dividing by 88.4 [15]. A preliminary version of the analysis applied creatinine in µmol/L directly to the mg/dL equation, producing values approximately 100-fold too low. This error was identified during the data audit described in Section 2.2 and corrected before any inferential model reported in this manuscript was fitted; every renal-function result presented here derives from the corrected values, and the correction is verified graphically in Supplementary Figure S1.

Body mass index (BMI) was calculated as weight in kilograms divided by height in metres squared. An audit of anthropometric data found zero values recorded for height and weight and a weight entry of 7214 kg, indicating data-entry rather than measurement error. Height outside 120–210 cm (553 values), weight outside 25–250 kg (859 values) and BMI outside 12–60 kg/m^2^ (65 values) were set to missing. Comorbidities were identified from Systematized Nomenclature of Medicine Clinical Terms (SNOMED CT)-coded diagnoses using pre-specified keyword patterns, generating binary flags for chronic kidney disease (CKD), diabetes mellitus (DM), hypertension (HTN), asthma, dyslipidaemia, renal transplantation, iron-deficiency anaemia and osteoarthritis; the comorbidity burden score is their sum.

### 2.6. Statistical Analysis

#### 2.6.1. Trajectory Analysis

Twenty linear mixed-effects models (four sites × five biomarkers) tested time × standardised biomarker interactions with patient-level random intercepts, adjusted for age, sex, BMI, comorbidity burden and flags for diabetes, hypertension and chronic kidney disease. Estimation was by restricted maximum likelihood (REML). Benjamini–Hochberg correction was applied across all twenty interaction tests [16]. Two further steps were pre-specified for interpreting any interaction reaching raw *p* < 0.05 without surviving correction. First, the observed number of such terms was compared with the number expected under the global null using an exact binomial test, since twenty independent tests at α = 0.05 yield one nominally significant result on average. Second, every coefficient was expressed as a percentage of the institutional least significant change per year, and as the number of years a one-standard-deviation (SD) difference in the biomarker would require to generate a divergence exceeding that threshold. A coefficient that cannot produce a detectable divergence within the observed follow-up period is not clinically interpretable irrespective of its *p*-value.

#### 2.6.2. Incident Osteoporosis

Time-to-event analysis is the primary approach. Cox proportional hazards regression was used: time zero is the baseline DXA at which the patient did not meet osteoporosis criteria, the event is the first subsequent DXA meeting those criteria, and patients without an event are censored at their last DXA. The proportional hazards assumption was tested with scaled Schoenfeld residuals. A secondary analysis using a fixed two-year window is reported in Supplementary Table S5, restricted to patients either followed for two years or having an event within two years (n = 586, 46 events), so that every patient contributes equivalent observation time. Ridge penalisation (λ = 0.05) was applied and fixed a priori rather than tuned, because no patient with normal baseline BMD experienced an event, which makes the maximum-likelihood estimate for that coefficient unbounded; cross-validation (CV) folds were stratified on event status.

Analyses were complete-case throughout. Chained-equation imputation is valid under a missing-at-random assumption, whereas measurement of vitamin D and ferritin here was driven by clinical suspicion and therefore depends on the unobserved value itself. With approximately 87–89% of values absent the fraction of missing information approaches unity and pooled estimates are governed by the imputation model rather than by the data. Retaining such output, even labelled as exploratory, invites it to be read as a finding; it has therefore been withdrawn rather than qualified.

### 2.7. Unmeasured Confounding

Anti-osteoporosis medication, glucocorticoid exposure, menopausal status and age at menopause, calcium and vitamin D supplementation, prior fragility fracture, physical activity, and bone turnover markers were unavailable. The absence of treatment data is the single most consequential limitation. In a cohort where a substantial proportion of osteopenic and osteoporotic patients would ordinarily receive antiresorptive therapy, an observed BMD slope reflects treatment response as much as natural history, and the direction of bias cannot be signed.

## 3. Results

### 3.1. Cohort Derivation and Attrition

Attrition across the analytic stages is substantial and central to interpretation. Of 9904 patients with any DXA examination, 2318 (23.4%) had two or more scans; 2147 (21.7% of the original cohort) met the one-year minimum interval and form the trajectory cohort; and of these, 789 (8.0% of the original cohort) did not have osteoporosis at baseline and form the incident-risk cohort. Each step selects for patients under active surveillance. Findings apply to the surveyed population and should not be extrapolated to unscreened community populations. The revised participant flow diagram (Supplementary Figure S2) specifies eligibility, time zero, outcome definition, censoring and every exclusion count.

The trajectory cohort comprised 2147 patients contributing 5674 DXA examinations, with a mean follow-up span of 2.77 years (range 1.00–4.93) and a mean of 2.64 scans per patient. Strata were stable or improved (n = 1441; 67.1%), moderate loss (n = 444; 20.7%) and rapid loss (n = 262; 12.2%). The 159 serial-scan patients excluded by the one-year rule differed from those retained only in sex distribution and, by construction, in follow-up span; their baseline characteristics are given in Supplementary Table S3.

### 3.2. Diagnostic Classification by Skeletal Site

Table 1 reports classification at each site. Osteoporosis prevalence varied several-fold across sites: 31.7% at the lumbar spine, 16.7% at the radius, 8.7% at the right femoral neck and 7.9% at the left femoral neck. Using the lowest T-score across all sites, as WHO and ISCD criteria specify, 39.1% of examinations met osteoporosis criteria. The lumbar spine supplied the lowest T-score in 69.0% of osteoporotic classifications and the radius in 21.2%. Because lumbar spine measurements are inflated by degenerative change in older patients, and because total hip was unavailable, the diagnostic label in this cohort is less femur-weighted than in cohorts where total hip is the reference site.

### 3.3. Baseline Characteristics by Trajectory Stratum

**Figure 1:**
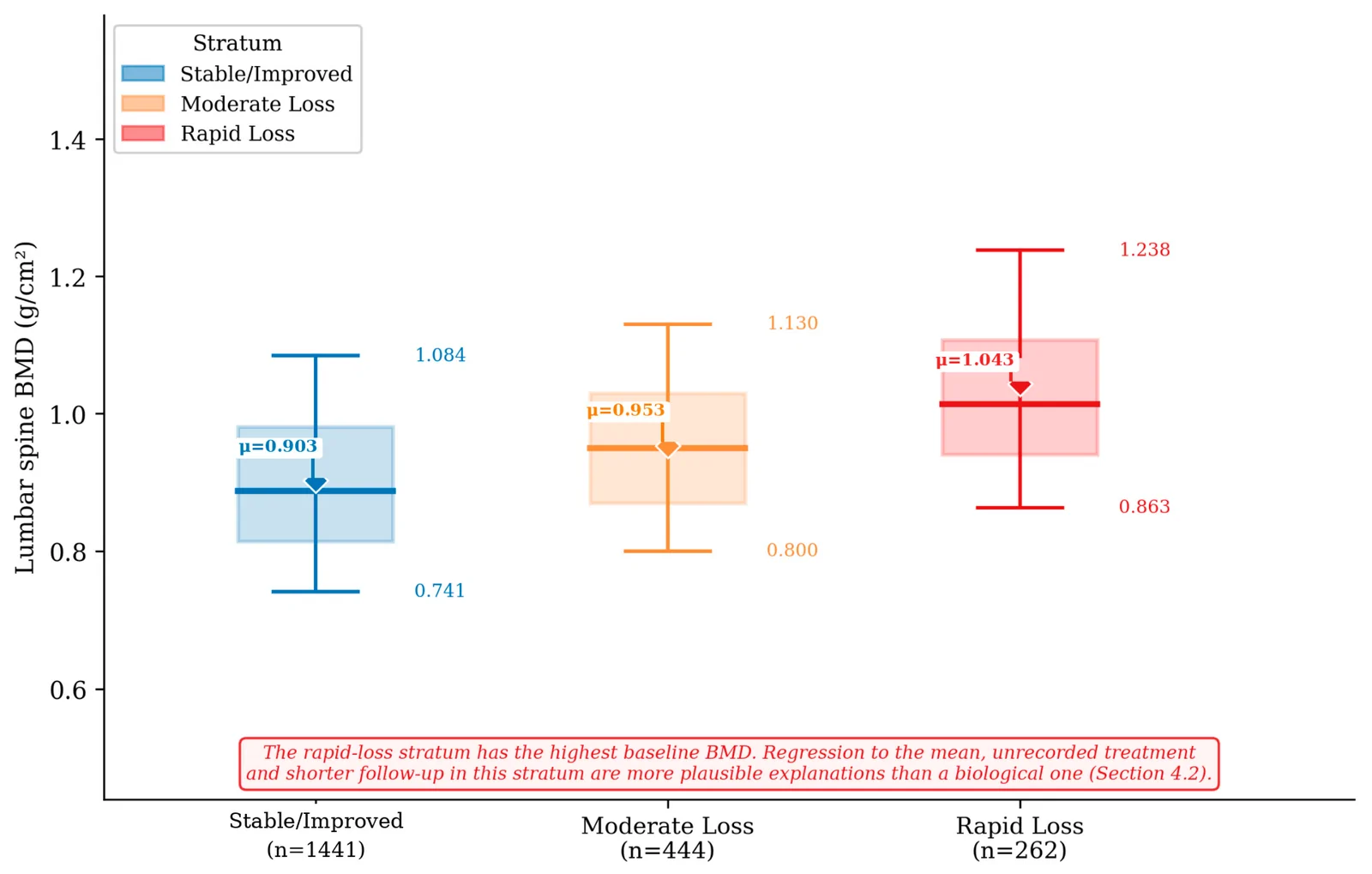
Baseline lumbar spine BMD by slope-derived trajectory stratum. Serial-scan patients with ≥1-year interval (n = 2147).

Age, sex, BMI and all baseline DXA measures differed across strata, and the direction of the BMD differences was counter-intuitive: the rapid-loss stratum had the highest baseline lumbar BMD (1.043 g/cm^2^) and the stable stratum the lowest (0.903 g/cm^2^); see Figure 1 and Table 2. Three explanations are more plausible than a biological one. Regression to the mean is expected whenever patients are stratified on a slope computed from the same measurements that provide the baseline value, and will by construction place higher baseline values in the declining stratum. Treatment is unrecorded, and patients with the lowest baseline BMD are those most likely to have started antiresorptive therapy and therefore to show stability or gain. Selection also contributes, since the rapid-loss stratum had a shorter mean follow-up span (2.40 versus 2.82 years) and fewer scans per patient. This pattern should not be read as showing that higher BMD causes faster loss.

**Figure 2:**
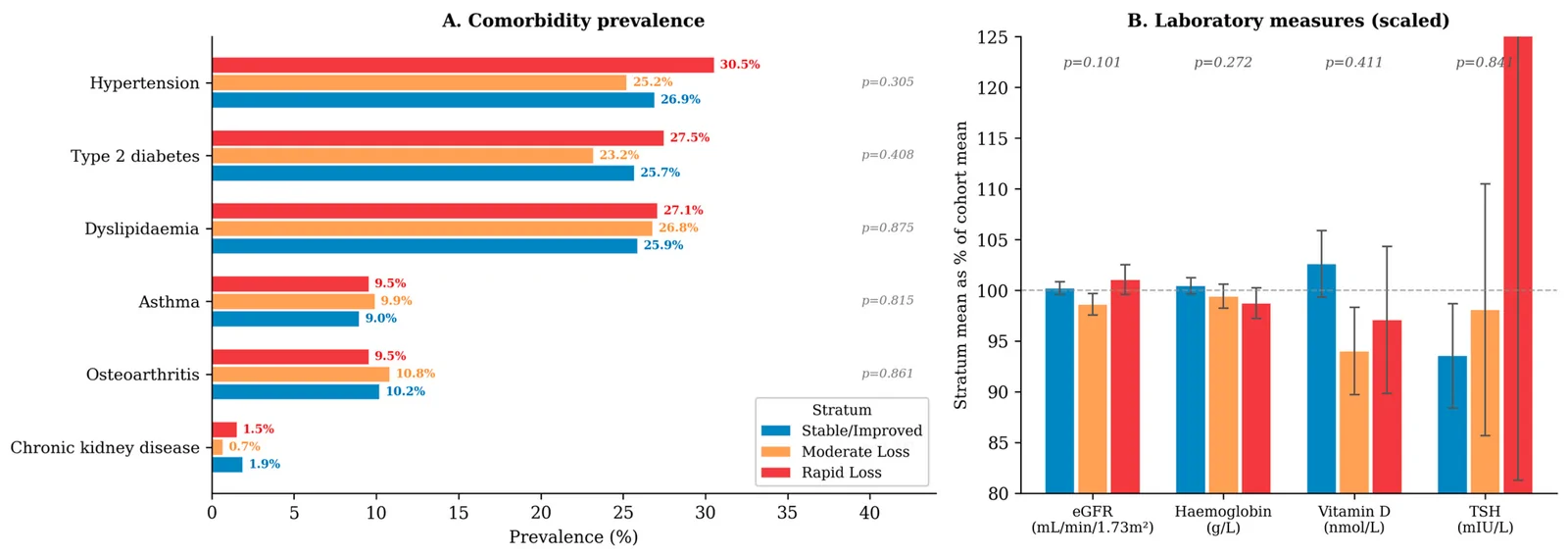
No comorbidity or laboratory measure differs across slope-derived trajectory strata (n = 2147).

No laboratory measure and no comorbidity differed across trajectory strata: eGFR (*p* = 0.101), haemoglobin (*p* = 0.272), vitamin D (*p* = 0.411), ferritin (*p* = 0.359), TSH (*p* = 0.841), comorbidity burden (*p* = 0.421), diabetes (*p* = 0.405), hypertension (*p* = 0.306) and chronic kidney disease (*p* = 0.210) (see Figure 2).

### 3.4. Time-to-Event Analysis of Incident Osteoporosis

Among 789 patients without osteoporosis at baseline followed for 2045 person-years, 107 developed osteoporosis (13.6%; 5.2 events per 100 person-years). The separation by baseline category was complete: no patient with normal baseline BMD progressed (0 of 124), whereas 107 of 665 with baseline osteopenia did (16.1%; log-rank *p* < 0.001). This magnitude of separation is partly definitional. A patient with a normal lowest T-score must cross more than 1.5 T-score units to meet osteoporosis criteria, whereas an osteopenic patient may need to cross only a fraction of one unit, and both are measured with the same instrument error.

In the Cox model (n = 384 with complete covariates, 53 events), baseline osteopenia carried the largest hazard ratio (3.12) but did not reach significance (95% CI 0.89–10.97; *p* = 0.076), and no other covariate was associated with progression (Table 3). The C-index was 0.679. The complete-case requirement reduced the analysis from 789 to 384 patients and from 107 to 53 events, giving fewer than seven events per covariate. Effect estimates from this model are unstable and the confidence intervals wide. The proportional hazards assumption held for every covariate (all Schoenfeld *p* > 0.13).

### 3.5. Trajectory Models

No time × biomarker interaction was significant after Benjamini–Hochberg correction; the smallest adjusted value was q = 0.214, and the conclusion was consistent across all four skeletal sites. Two of the twenty interaction terms reached raw *p* < 0.05 (ferritin at the right femoral neck, *p* = 0.019; vitamin D at the right femoral neck, *p* = 0.021) and two more fell between 0.05 and 0.10. Twenty independent tests at α = 0.05 yield one nominally significant result on average, so observing two is unremarkable (exact binomial *p* = 0.26 against the global null); the four results below *p* = 0.10 are likewise within chance expectation (2.0 expected; *p* = 0.13). These terms are reported for completeness and are not interpreted as signals.

Their magnitude reinforces that reading. Table 4 expresses each coefficient against the institutional least significant change of 0.033 g/cm^2^. The largest, vitamin D at the right femoral neck, corresponds to 15.0% of the least significant change per year: a one-standard-deviation difference in baseline vitamin D—35 nmol/L, spanning most of the clinically encountered range—would require 6.7 years to generate a divergence that the densitometer could resolve, against an observed mean follow-up of 2.77 years. The ferritin coefficient would require 10.7 years. None of these effects could be detected in an individual patient within the study period, and none approaches a magnitude at which monitoring would alter management. Directionality is also inconsistent: the ferritin coefficient is positive at the lumbar spine and negative at both cortical-dominant sites, which is the pattern expected of noise rather than of a systemic effect on bone.

## 4. Discussion

This cohort addressed two distinct questions and returned a consistent answer to both. Baseline DXA category identified who progressed to osteoporosis; no routine laboratory measure was associated with either the rate of BMD change or the risk of progression. The two analyses are complementary rather than redundant: the trajectory models ask whether a biomarker bends a continuous curve, whereas the time-to-event analysis asks whether a baseline state predicts crossing a clinical threshold. A marker could in principle do one without the other; here none did either.

### 4.1. Comparison with Previous Longitudinal Studies

The finding that no patient with normal baseline BMD progressed over a mean of 2.6 years is consistent with the largest longitudinal analysis of this question. In the Study of Osteoporotic Fractures, Gourlay and colleagues followed 4957 women aged 67 or older for up to 15 years and estimated the interval for 10% to develop osteoporosis at 16.8 years for those with normal BMD and 17.3 years for mild osteopenia, falling to 4.7 years for moderate and 1.1 years for advanced osteopenia [17]. A Korean cohort reported closely comparable intervals of 13.2, 5.0 and 1.5 years across mild, moderate and severe osteopenia [18]. Against those benchmarks, an observation period of 2.6 years is far too short for transitions from normal BMD to be expected, and the zero events in that stratum are the anticipated result rather than a novel one.

Regarding biomarkers, the most informative longitudinal comparisons come from cohorts with repeated marker measurement. In the Study of Women’s Health Across the Nation, within-woman increases in C-reactive protein (CRP) predicted subsequent acceleration of BMD loss in 1431 women, using individual fixed-effects models adjusted for menopausal stage and bone-active medication [19]—a design that isolates within-person change and controls for treatment, neither of which was possible here. Studies that do detect biomarker effects on BMD change have generally measured bone turnover markers directly [20], and it is the turnover markers rather than the general metabolic panel that carry the signal. The present null result should therefore be read as applying to the routine chemistry panel obtained incidentally around a DXA appointment, not to skeletal biomarkers as a class.

Prediction-model comparisons are similarly instructive. Published osteoporosis prediction models achieve areas under the curve of roughly 0.70–0.85, but almost all predict prevalent osteoporosis cross-sectionally and many include BMD or T-score among the predictors, which inflates apparent performance [21]. Models predicting incident disease over a defined horizon perform considerably less well, and the C-index of 0.679 obtained here sits within that more modest range.

### 4.2. Baseline BMD and Subsequent Slope

The inverse relation between baseline BMD and subsequent slope is most plausibly explained by regression to the mean, unrecorded treatment and differential follow-up length, as set out in Section 3.3. This pattern is a general hazard of stratifying patients on a slope derived from the same series that supplies the baseline value, and it argues for anchoring trajectory definitions to the least significant change rather than to distributional cutoffs.

### 4.3. Skeletal Site and Diagnostic Definition

The several-fold variation in osteoporosis prevalence across sites (7.9% to 31.7%) illustrates that a single-site definition would have produced a materially different cohort. The absence of total hip measurement is a genuine constraint: the total hip has the lowest precision error of any site, is the reference site for FRAX calibration, and is the site least affected by degenerative artefact. Its unavailability means the diagnostic label here rests disproportionately on the lumbar spine, where osteophytosis and aortic calcification inflate BMD in exactly the older patients most at risk, and on the radius, which is not recommended for routine diagnosis except when spine and hip are unmeasurable.

### 4.4. Interpretation of the Borderline Interaction Terms

Four of the twenty interaction terms fell below *p* = 0.10 before correction and two below *p* = 0.05, involving vitamin D, ferritin and TSH. We do not regard these as findings and they are not offered as a contribution of this study, for four reasons that are individually sufficient. They do not survive correction for the twenty tests performed, with the smallest adjusted value of q = 0.214. Their number is what chance alone predicts, and an exact binomial test gives no evidence against the global null. Their magnitude is below the threshold at which the densitometer used in this study can resolve a difference: each would require between 6.7 and 13.9 years to generate a divergence exceeding the least significant change, against an observed follow-up of 2.77 years. And the analytes concerned were measured in only 11–13% of the cohort, in selected patients because a clinician suspected an abnormality, so even a real association could not be distinguished from confounding by indication.

The wider point concerns how such results should be handled. A study performing twenty tests will ordinarily produce one or two nominally significant results whatever the truth, and reporting these as candidate signals—however carefully hedged—contributes to a literature in which underpowered biomarker associations accumulate without replication [22]. The appropriate treatment is to report them transparently, quantify their magnitude against a measurement-based threshold, and decline to interpret them. That is the approach taken here, and it is why the multiple-imputation sensitivity analysis has been withdrawn entirely rather than retained with qualifications.

### 4.5. Missing Data

Vitamin D and ferritin were missing in roughly 87–89% of patients, and the missingness is not random: patients were tested because a clinician suspected deficiency or was investigating anaemia or renal disease. Complete-case analysis is used throughout, and its limitation—that the analysed subset is a clinically selected sample in whom abnormality was suspected—is stated rather than addressed by imputation. Multiple imputation was considered and rejected: chained-equation methods assume data are missing at random, which is not the mechanism here, and at this level of missingness the imputation model rather than the data would determine the result.

### 4.6. Strengths

Trajectories were derived from serial measurements rather than inferred from baseline diagnosis; the extraction algorithm was audited against manually abstracted values with the audit worksheet provided; multiple testing was controlled across all twenty interaction models; and the diagnostic definition follows WHO and ISCD criteria using the lowest of four measured sites.

### 4.7. Limitations

Anti-osteoporosis treatment, glucocorticoid exposure, menopausal status, supplementation, prior fracture and bone turnover markers were unavailable, and treatment absence in particular means observed slopes conflate response with natural history. Total hip BMD was not measured. Vitamin D and ferritin were missing in most patients. The complete-case Cox model rests on 53 events. Follow-up averaged 2.8 years, short relative to the intervals over which transitions from normal BMD occur. The cohort is drawn from a single tertiary centre and is selected by referral for DXA.

### 4.8. Future Directions

Prospective cohorts in this region should record anti-osteoporosis prescriptions with start and stop dates, glucocorticoid exposure, menopausal status and prior fracture, add total hip acquisition, and measure procollagen type 1 N-terminal propeptide (P1NP) and β-CTX (Beta C-terminal telopeptide of type I collagen) alongside routine chemistry. Treatment must be tracked as a time-varying exposure rather than a baseline covariate, and any model must be validated externally before clinical use. The present findings do not support prioritising vitamin D or ferritin as candidate biomarkers for monitoring BMD trajectories. Their inclusion in future bone-health research should therefore be guided by established clinical indications and biological rationale rather than by associations observed in the current study.

## 5. Conclusions

In this longitudinal Saudi DXA cohort, baseline DXA category distinguished patients who progressed to osteoporosis, while routine laboratory measures were not associated with either BMD trajectory or progression after adjustment. No patient with normal baseline bone density progressed within a mean of 2.6 years, consistent with established estimates of transition intervals. No biomarker examined here has demonstrated clinical utility for monitoring bone loss, and none is proposed as a candidate for that purpose. The contribution of this work is a carefully specified null result in a large real-world cohort—obtained after correcting several data-integrity defects, restricting trajectory analysis to measurements far enough apart to exceed measurement error, and controlling for multiple testing—together with the reminder that baseline densitometric category remains the practical basis for deciding whom to re-scan.

## Figures and Tables

**Table 1:** Diagnostic classification by skeletal site (all examinations).

Skeletal Site	n Scans	Normal (T > −1.0)	Osteopenia (−2.5 < T ≤ −1.0)	Osteoporosis (T ≤ −2.5)	% Driving Lowest-T ^a^
Lumbar spine (L1–L4)	13,112	3158 (24.1%)	5800 (44.2%)	4154 (31.7%)	69.0%
Femoral neck—left	12,969	6672 (51.4%)	5266 (40.6%)	1031 (7.9%)	5.0%
Femoral neck—right	12,947	6381 (49.3%)	5440 (42.0%)	1126 (8.7%)	4.8%
Distal radius	10,579	5194 (49.1%)	3619 (34.2%)	1766 (16.7%)	21.2%
Lowest T-score, any site	13,147	2092 (15.9%)	5915 (45.0%)	5140 (39.1%)	—
Total hip	4 (0.03%)	Not acquired at this institution—see Section 2.3			

^a^ Percentage of osteoporotic classifications for which that site supplied the lowest T-score. Percentages within each site row are of examinations with a valid T-score at that site; denominators differ because not all sites were measured in all examinations.

**Table 2:** Baseline characteristics by BMD trajectory stratum (serial-scan patients with ≥1-year interval).

Variable	Overall (n = 2147)	Stable/Improved (n = 1441)	Moderate Loss (n = 444)	Rapid Loss (n = 262)	*p*
**Demographics**					
Age (years)	59.2 ± 13.7	59.3 ± 14.5	59.9 ± 11.9	57.7 ± 11.6	<0.001
Female sex, n (%)	1900 (88.5%)	1254 (87.0%)	413 (93.0%)	233 (88.9%)	0.002
BMI (kg/m^2^)	29.92 ± 6.43	29.62 ± 6.25	30.06 ± 6.60	31.28 ± 6.90	0.011
**DXA measurements and trajectory metrics**					
Lumbar spine BMD (g/cm^2^)	0.930 ± 0.161	0.903 ± 0.147	0.953 ± 0.130	1.043 ± 0.213	<0.001
Lumbar T-score	−2.16 ± 1.19	−2.37 ± 1.16	−1.92 ± 1.03	−1.44 ± 1.22	<0.001
Lowest T-score, any site ^a^	−2.43 ± 1.06	−2.62 ± 0.99	−2.19 ± 0.99	−1.78 ± 1.16	<0.001
Annual BMD slope (g/cm^2^/yr)	+0.004 ± 0.047	+0.017 ± 0.040	−0.012 ± 0.004	−0.044 ± 0.073	<0.001
Follow-up span (years) ^b^	2.77 ± 0.97	2.82 ± 0.97	2.84 ± 0.96	2.40 ± 0.91	<0.001
DXA scans per patient	2.64 ± 0.91	2.71 ± 0.94	2.58 ± 0.87	2.34 ± 0.69	<0.001
FRAX major fracture (%)	10.44 ± 4.68	10.86 ± 4.84	10.27 ± 4.68	9.38 ± 3.92	0.005
FRAX hip fracture (%)	1.40 ± 1.62	1.60 ± 1.90	1.25 ± 1.20	1.00 ± 1.01	<0.001
**Laboratory Parameters**					
eGFR (mL/min/1.73 m^2^) ^c^	95.8 ± 21.3	96.0 ± 21.5	94.5 ± 20.2	96.8 ± 21.4	0.101
Haemoglobin (g/L) ^c^	128.0 ± 14.9	128.6 ± 15.8	127.3 ± 12.6	126.4 ± 13.9	0.272
Vitamin D 25-OH (nmol/L) ^c^	81.9 ± 35.1	84.0 ± 36.8	77.0 ± 28.0	79.5 ± 36.6	0.411
Ferritin (µg/L) ^c^	152.7 ± 498.0	185.9 ± 619.1	79.6 ± 109.8	118.5 ± 156.7	0.359
TSH (mIU/L) ^c^	3.30 ± 8.19	3.09 ± 4.21	3.24 ± 5.64	4.59 ± 20.09	0.841
**Comorbidities**					
Comorbidity burden score	1.03 ± 1.32	1.03 ± 1.32	0.99 ± 1.31	1.10 ± 1.33	0.421
Diabetes mellitus, n (%)	545 (25.4%)	370 (25.7%)	103 (23.2%)	72 (27.5%)	0.405
Hypertension, n (%)	580 (27.0%)	389 (27.0%)	112 (25.2%)	79 (30.2%)	0.306
Dyslipidaemia, n (%)	562 (26.2%)	373 (25.9%)	119 (26.8%)	70 (26.7%)	0.883
Osteoarthritis, n (%)	221 (10.3%)	148 (10.3%)	48 (10.8%)	25 (9.5%)	0.865
Chronic kidney disease, n (%)	34 (1.6%)	27 (1.9%)	3 (0.7%)	4 (1.5%)	0.210

Height, weight and BMI values outside physiologically plausible ranges were set to missing before analysis (Methods 2.5). ^a^ Lowest T-score across lumbar spine, both femoral necks and distal radius. ^b^ Interval between first and last DXA examination. ^c^ Available in a subset: eGFR n = 1896 (88.3%), TSH n = 917 (42.7%), haemoglobin n = 357 (16.6%), vitamin D n = 288 (13.4%), ferritin n = 240 (11.2%). Continuous variables compared by Kruskal–Wallis test, categorical by chi-squared test.

**Table 3:** Cox proportional hazards model for incident osteoporosis (primary analysis).

Predictor	β	HR	95% CI	*p*	Schoenfeld *p*
Baseline osteopenia (vs normal)	1.138	3.120	0.888–10.967	0.076	0.980
BMI (per kg/m^2^)	0.040	1.041	0.994–1.089	0.090	0.134
Female sex	0.653	1.921	0.544–6.785	0.311	0.570
Diabetes mellitus	0.191	1.211	0.634–2.313	0.563	0.712
Age (per year)	−0.013	0.987	0.954–1.020	0.438	0.737
FRAX major fracture (%)	−0.040	0.961	0.909–1.016	0.160	0.582
Comorbidity burden	−0.095	0.909	0.711–1.163	0.449	0.348
Hypertension	−0.321	0.725	0.391–1.346	0.308	0.869

**Table 4:** Magnitude of the four interaction terms with raw *p* < 0.10, expressed against the institutional least significant change.

Biomarker (Site)	β per SD (g/cm^2^/yr)	Raw *p*	q (FDR)	% Of LSC Per Year	Years to Reach LSC	Detectable Within Study Follow-Up?
Ferritin (FN-right)	−0.00309	0.019	0.214	9.4%	10.7	No
Vitamin D (FN-right)	+0.00496	0.021	0.214	15.0%	6.7	No
Vitamin D (FN-left)	+0.00374	0.096	0.411	11.3%	8.8	No
TSH (radius)	−0.00237	0.099	0.411	7.2%	13.9	No

False discovery rate, (FDR) LSC = least significant change at 95% confidence for lumbar spine BMD at this institution (0.033 g/cm^2^). “Years to reach LSC” is the time a one-standard-deviation difference in the biomarker would need to generate a BMD divergence exceeding the LSC. The mean observed follow-up was 2.77 years, shorter than every value in that column. q values are Benjamini–Hochberg adjusted across all 20 interaction tests.

## Data Availability

The datasets presented in this article are not readily available because the data are part of an ongoing study. Requests to access the datasets should be directed to the corresponding author.

## Supplementary Materials

The following supporting information can be downloaded at: https://www.mdpi.com/article/10.3390/jcm15176581/s1, Figure S1, eGFR Unit Error Verification and Correction; Figure S2, Participant flow and analytic populations; Table S1, completed STROBE checklist; Table S2, DXA narrative extraction algorithm and audit; Table S3, Sensitivity to the minimum inter-scan interval; Table S4, Sensitivity to BMD slope cutoff definitions; Table S5, Fixed two-year window analysis.

## Abbreviations

The following abbreviations are used in this manuscript:

25-OH vitamin D	25-hydroxyvitamin D
BMD	Bone mineral density
BMI	Body mass index
CI	Confidence interval
CKD	Chronic kidney disease
CKD-EPI	Chronic Kidney Disease Epidemiology Collaboration
CRP	C-reactive protein
CV	Cross-validation
DM	Diabetes mellitus
DXA	Dual-energy X-ray absorptiometry
eGFR	Estimated glomerular filtration rate
FDR	False discovery rate
FN-L	Left femoral neck
FN-R	Right femoral neck
FRAX	Fracture Risk Assessment Tool
HR	Hazard ratio
HTN	Hypertension
KSUMC	King Saud University Medical City
LME	Linear mixed-effects
LSC	Least significant change
OLS	Ordinary least squares
P1NP PH	Procollagen type 1 N-terminal propeptide Proportional hazards
PTH	Parathyroid hormone
RBC	Red blood cell count
REML	Restricted maximum likelihood
RT	Right
SD	Standard deviation
SE	Standard error
SNOMED CT	Systematised Nomenclature of Medicine Clinical Terms
STROBE	Strengthening the Reporting of Observational Studies in Epidemiology
T2DM	Type 2 diabetes mellitus
TBS	Trabecular bone score
TSH	Thyroid-stimulating hormone
VIF	Variance inflation factor
WHO	World Health Organization
β-CTX	Beta C-terminal telopeptide of type I collagen
