# Supplementary material for: Clinical and Laboratory Predictors of Bone Mineral Density Trajectories and Osteoporosis Progression: A Retrospective Longitudinal Cohort Study
Source: J Clin Med. 2026 Aug 26;15(17):6581. doi: 10.3390/jcm15176581 (PMC13566557; doi:10.3390/jcm15176581)

Table S1. STROBE checklist

Checklist for cohort studies (Vandenbroucke et al., PLoS Med 2007;4(10):e297). Locations refer to the revised manuscript. Section numbers reflect the corrected Discussion numbering (4.1–4.7).

No.	Section	Recommendation	Reported	Location in revised manuscript
**Title and abstract**				
1a	**Title**	Indicate the study's design with a commonly used term in the title or abstract	Yes	Title
1b	**Abstract**	Provide an informative and balanced summary of what was done and what was found	Yes	Abstract
**Introduction**				
2	**Background**	Explain the scientific background and rationale for the investigation	Yes	Section 1, ¶1–3
3	**Objectives**	State specific objectives, including any prespecified hypotheses	Yes	Section 1, ¶4
**Methods**				
4	**Study design**	Present key elements of study design early in the paper	Yes	Section 2.1
5	**Setting**	Describe setting, locations, and relevant dates including recruitment and follow-up	Yes	Section 2.1
6a	**Participants**	Give eligibility criteria and sources/methods of selection; describe follow-up	Yes	Sections 2.4, 3.1
6b	**Matching**	For matched studies, give matching criteria and number of exposed/unexposed	NA	Not a matched design
7	**Variables**	Define all outcomes, exposures, predictors, confounders, effect modifiers	Yes	Sections 2.3–2.5
8	**Data sources**	For each variable give sources of data and details of methods of assessment	Yes	Sections 2.2–2.5; S5
9	**Bias**	Describe any efforts to address potential sources of bias	Yes	Sections 2.2, 2.7, 4.6
10	**Study size**	Explain how the study size was arrived at	Yes	Section 3.1; Figure S2
11	**Quantitative variables**	Explain how quantitative variables were handled; describe groupings and why	Yes	Section 2.4; Table S7
12a	**Statistical methods**	Describe all statistical methods including those to control confounding	Yes	Section 2.6
12b	**Subgroups**	Describe methods used to examine subgroups and interactions	Yes	Section 2.6.1
12c	**Missing data**	Explain how missing data were addressed	Yes	Sections 2.6, 4.4; Table S2
12d	**Loss to follow-up**	Explain how loss to follow-up was addressed	Yes	Section 2.6.2 (censoring)
12e	**Sensitivity analyses**	Describe any sensitivity analyses	Yes	Tables S6, S7, S8
**Results**				
13a	**Participant flow**	Report numbers of individuals at each stage of the study	Yes	Section 3.1; Figure S2
13b	**Non-participation**	Give reasons for non-participation at each stage	Yes	Figure S2; Table S6

No.	Section	Recommendation	Reported	Location in revised manuscript
13c	**Flow diagram**	Consider use of a flow diagram	Yes	Figure S2
14a	**Descriptive data**	Give characteristics of participants and information on exposures/confounders	Yes	Table 1
14b	**Missing per variable**	Indicate number of participants with missing data for each variable	Yes	Table 1 footnote; Table S2
14c	**Follow-up time**	Summarise follow-up time	Yes	Sections 3.1, 3.4
15	**Outcome data**	Report numbers of outcome events or summary measures over time	Yes	Sections 3.4–3.5; Table 3
16a	**Main results**	Give unadjusted and confounder-adjusted estimates with precision	Yes	Tables 1–3
16b	**Category boundaries**	Report category boundaries when continuous variables were categorised	Yes	Sections 2.3–2.4
16c	**Absolute risk**	Consider translating relative risk into absolute risk for a meaningful period	Yes	Section 3.4 (rate per 100 PY)
17	**Other analyses**	Report other analyses done — subgroups, interactions, sensitivity analyses	Yes	Section 3.5; Tables S6–S8
**Discussion**				
18	**Key results**	Summarise key results with reference to study objectives	Yes	Section 4, ¶1
19	**Limitations**	Discuss limitations, sources of bias or imprecision; direction and magnitude	Yes	Section 4.6
20	**Interpretation**	Give a cautious overall interpretation considering objectives, limitations, multiplicity	Yes	Sections 4.1–4.4; 5
21	**Generalisability**	Discuss the generalisability (external validity) of the study results	Yes	Sections 3.1, 4.6
**Other information**				
22	**Funding**	Give the source of funding and the role of the funders	Yes	Declarations

Table S2. DXA narrative extraction algorithm and audit

S2.1 Source format

DXA results are stored as free-text radiologist narratives. A representative record, with identifiers removed:

```
NM BONE DENSITY SCAN DXA of 30-DEC-2021:

Lumbar Spine BMD = 0.903  g/cm  (-2.4  T-SCORE)    (-1.5  Z-SCORE)
Femoral Neck BMD = 0.791  g/cm  (-1.7  T-SCORE)    (-1.1  Z-SCORE) LT
Femoral Neck BMD = 0.761  g/cm  (-2    T-SCORE)    (-1.3  Z-SCORE) RT
Radius BMD       = 0.871  g/cm  (-0.1  T-SCORE)    ( 0.4  Z-SCORE)

IMPRESSION:
Osteopenia
```

Laterality appears as a trailing token (LT / RT) rather than in the label, which is the feature the original pipeline failed to use and the reason the left femoral neck value was propagated to the right femoral neck and to the radius.

S2.2 Extraction patterns

Each site is matched independently; no value is inherited from another site.

```
lumbar    :  /[Ll]umbar[^\n]*BMD[^\n]*/
femoral   :  /[Ff]emoral[^\n]*BMD[^\n]*/     then split on /\bLT\b|left/i
                                              and  /\bRT\b|right/i
radius    :  /[Rr]adius[^\n]*BMD[^\n]*/
BMD value:  /=\s*([\d]*\.?[\d]+)/           accept only 0.1 < x < 3.0 g/cm²
T-score   :  /\(\s*(-?[\d]*\.?[\d]+)\s*T/i  accept only -8.0 < x < 8.0
```

Values outside the stated ranges are set to missing rather than retained, which is why site denominators differ.

S2.3 Audit procedure and results

Stage 1: 100 reports were selected at random and abstracted manually by a reader blinded to the algorithm output. Agreement was scored on exact numeric match to three decimal places, not on a tolerance band. Stage 2: every parsed BMD was checked for internal consistency against the parsed T-score for the same site; records failing this check were re-parsed.

Site	Records parsed	Exact-match agreement	Discordant	Cause of discordance
Lumbar spine	13,112	**98.0%**	2	Non-standard line format
Femoral neck — left	12,969	**97.0%**	3	Missing laterality token
Femoral neck — right	12,947	**96.0%**	4	Missing laterality token
Distal radius	10,579	**94.0%**	6	Tab-delimited value column

No discordance arose from numeric misreading; all were formatting variants. Two defects were identified through this audit and corrected before any inferential model reported in the manuscript was fitted: propagation of the left femoral neck BMD to the other non-lumbar sites, and the same propagation affecting site-specific T-scores. All 13,626 narratives were re-parsed after correction.

Table S3. Sensitivity to the minimum inter-scan interval

With a least significant change of 0.033 g/cm², an interval of a few weeks cannot separate true change from measurement error, and annualising such an interval multiplies that error. The table shows the consequence: shortening the permitted interval increases the number of patients with a biologically implausible slope. The one-year threshold was adopted.

Minimum interval	N	Stable	Moderate	Rapid	Mean slope ± SD	|β|>0.10	Median span (yr)
None (original)	2,306	1,540	463	303	+0.0039 ± 0.0498	57	2.65
≥6 months	2,278	1,524	461	293	+0.0043 ± 0.0475	46	2.68
**≥1 year (adopted)**	**2,147**	**1,441**	**444**	**262**	**+0.0038 ± 0.0469**	**33**	**2.74**
≥2 years	1,591	1,084	347	160	+0.0026 ± 0.0378	18	3.05

|β|>0.10 counts patients whose fitted annual slope exceeds 0.10 g/cm²/yr in absolute value — roughly three times the least significant change per year, and not biologically credible over the observed intervals. The adopted row is shaded.

Table S3b. Characteristics of patients excluded by the one-year rule

The 159 patients with serial scans but less than one year of total observation are compared with those retained. They differ from the retained cohort only in sex distribution and, by construction, in follow-up span; no baseline DXA or laboratory measure differs. Their exclusion therefore narrows the cohort toward patients under sustained surveillance without introducing an evident severity bias.

Baseline characteristic	Excluded (n=159)	Retained (n=2,147)	p	Difference
Age (years)	56.6 ± 17.9	59.2 ± 13.7	0.192	Not significant
BMI (kg/m²)	29.30 ± 7.21	29.92 ± 6.43	0.216	Not significant
Lumbar spine BMD (g/cm²)	0.945 ± 0.164	0.930 ± 0.161	0.142	Not significant
Lowest T-score, any site	−2.33 ± 1.16	−2.43 ± 1.06	0.312	Not significant
Comorbidity burden score	1.09 ± 1.50	1.03 ± 1.32	0.912	Not significant
Female sex	82.4%	88.5%	**0.010**	Fewer women excluded
Diabetes mellitus	29.6%	25.4%	1.000	Not significant
Follow-up span (years)	0.73 ± 0.24	2.77 ± 0.97	**<0.001**	By construction

Table S4. Sensitivity to BMD slope cutoff definitions

The primary cutoffs were chosen so that the moderate/rapid boundary approximates half the least significant change per year (0.0165 g/cm²/yr), meaning a patient classed as rapid loss would exceed the least significant change within approximately two years. Because the choice remains partly conventional, three alternatives were tested.

Scheme	Cutoffs (g/cm²/yr)	Stable	Moderate	Rapid	p eGFR	p Hgb	p VitD	p Age
**Primary**	**−0.005 / −0.020**	**1,441**	**444**	**262**	**0.101**	**0.272**	**0.411**	**<0.001**
Narrower	−0.003 / −0.015	1,357	409	383	0.737	0.608	0.482	<0.001
Wider	−0.010 / −0.030	1,602	420	127	0.684	0.368	0.374	<0.001
LSC-anchored	0 / −0.0165	1,204	607	338	0.455	0.348	0.893	<0.001

Stratum sizes shift as expected across schemes, but no laboratory comparison becomes significant under any alternative definition, and age remains significant under all four. The conclusion that laboratory measures do not differ across trajectory strata is not an artefact of the cutoff choice. The primary scheme is shaded.

Table S5. Fixed two-year window analysis

The primary incident analysis is Cox regression, which handles unequal observation time directly. This fixed-window analysis provides the alternative the reviewers proposed: patients are eligible only if followed for two years or if an event occurred within two years, so every patient contributes equivalent observation time.

Population	N	Events within 2 yr	Event rate	Comparison with primary
Eligible for fixed 2-year window	586	46	7.8%	—
Normal at baseline	88	0	0.0%	Matches primary (0/124)
Osteopenia at baseline	498	46	9.2%	Primary: 16.1% over full FU
Excluded: censored <2 yr, no event	203	—	—	Removes unequal observation

The zero event count among patients with normal baseline BMD is reproduced exactly in this restricted analysis (0 of 88), confirming that it is not an artefact of differential follow-up.

Table S5b. Penalised logistic regression within the two-year window

Ridge-penalised logistic regression (C = 0.1) on the 277 patients with complete covariate data and 20 events; five-fold cross-validated AUC 0.607. Every coefficient shares the direction of its Cox counterpart, which supports the primary analysis rather than adding to it.

Predictor	OR	Direction	Consistency with Cox model
Baseline osteopenia (vs normal)	1.261	↑	Same direction as Cox (HR 3.12); attenuated by penalisation
Female sex	1.234	↑	Same direction as Cox (HR 1.92)
BMI (per SD)	1.038	↑	Same direction as Cox (HR 1.04)
Diabetes mellitus	1.063	↑	Same direction as Cox (HR 1.21)
Hypertension	0.862	↓	Same direction as Cox (HR 0.73)
Comorbidity burden	0.766	↓	Same direction as Cox (HR 0.91)
Age (per SD)	0.839	↓	Same direction as Cox (HR 0.99)
FRAX major fracture (%)	0.735	↓	Same direction as Cox (HR 0.96)

With 20 events and eight covariates this model is severely overfitted and its coefficients should not be interpreted individually. It is presented solely to demonstrate that the choice between time-to-event and fixed-window framing does not alter the direction of any association. The cross-validated AUC of 0.607 is close to the Cox C-index of 0.679, both indicating modest discrimination.

Figure S1, eGFR Unit Error Verification and Correction

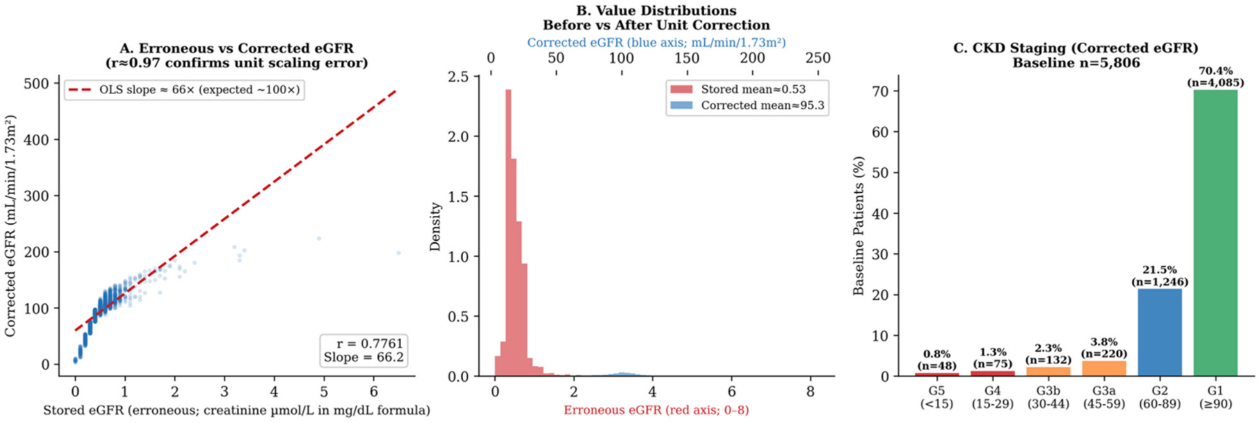

Figure S2, Participant flow and analytic populations

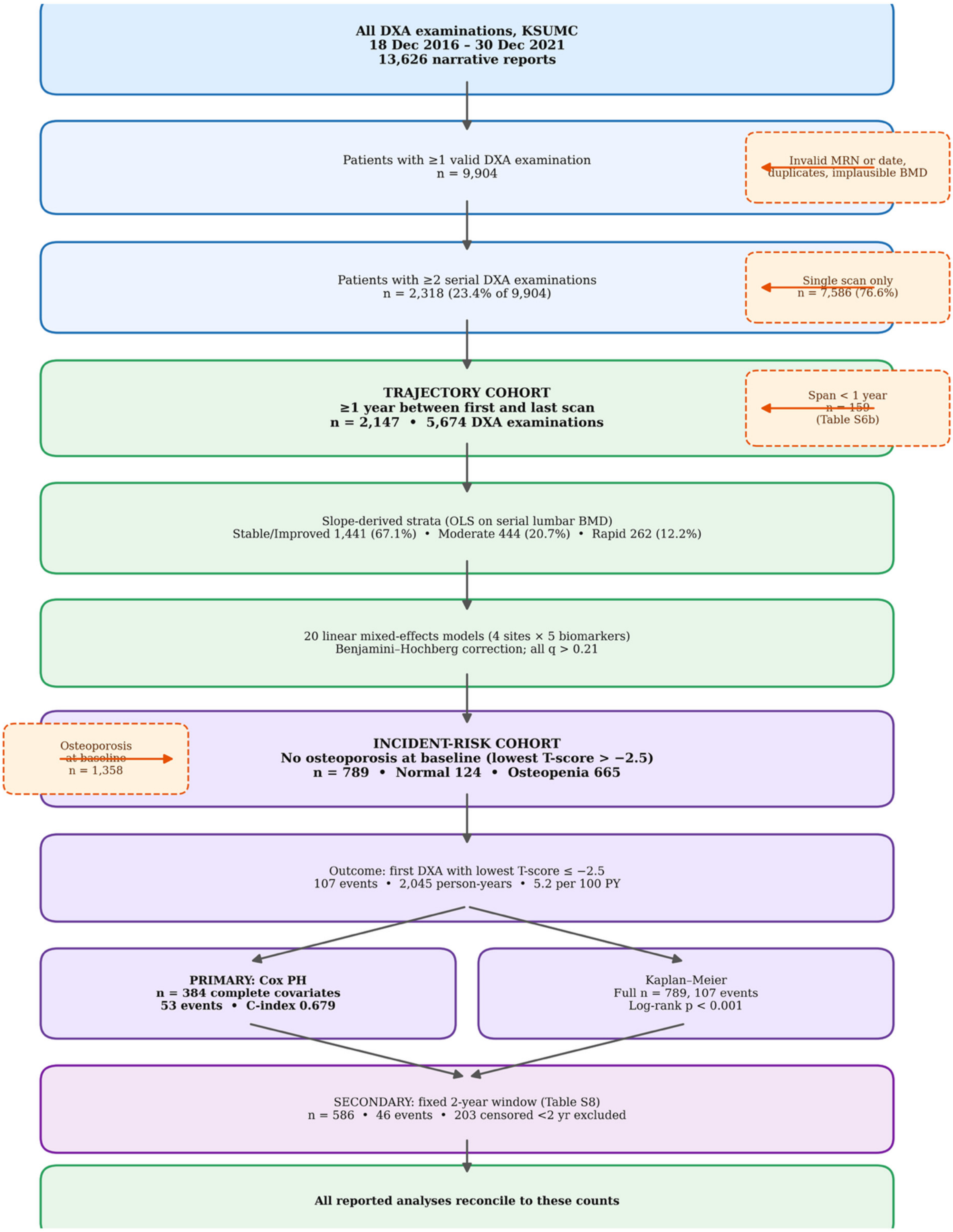

Supplement: Supplementary file 1 [file jcm-15-06581-s001.zip › jcm-4473162-supplementary.pdf]
